# Supplementary material for: Mapping the organisational network of support for people experiencing homelessness in rural coastal areas of North East England: Results from a mixed-methods multi-sector social network analysis
Source: PLOS Ment Health. 2024 Dec 20;1(7):e0000207. doi: 10.1371/journal.pmen.0000207 (PMC12798162; doi:10.1371/journal.pmen.0000207)
Supplement: S1 Text — (DOCX) [file pmen.0000207.s001.docx]

**Interview Topic Guide**

**Introduction**

- *Thank you for seeing me today and offering to take part in this study*
- *The interview/focus group will last approximately 60 minutes*
- *I would like to first outline the study so that you are able to decide whether you wish to proceed further (recap information sheet and study description)*
- *Participation is optional and you can stop the interview or decline to answer specific individual questions at any time should you wish*

**Confidentiality and Consent**

- Reiterate issues of confidentiality and anonymity, the purpose of the study and what is going to happen to the data
- **Verbally confirm that participants understand the purpose and confidentiality of the research, that they are happy to take part and that parts of the session will be audio recorded**
- Complete consent forms
- Ask if participants have any questions

**Study Description**

This interview is part of a research project where we are looking to gather information to help build a 'picture' of the network of organisations or community groups, that provide support or services to people experiencing homelessness* in Northumberland and North Tyneside. The information we are gathering from various groups will build a 'map' of services – to better understand the different local organisations and support structures for people experiencing homelessness as well as for staff providing that support. Ultimately, our research project is about ways to improve holistic support for people experiencing homelessness – and having a good understanding of the network of support across the system is a really important part of knowing where to get that holistic support from.

*Insecure housing, sleeping on a friend’s sofa, rough sleeping, staying in supported housing

Your data will be secure. Your personal information will remain anonymous and will not be shared. We will ask you to share information about other organisations we should connect with. Are you happy for us to use your name if/when we contact these people?

| **Objectives**   1. **Map and describe the range of support** for people experiencing or at risk of homelessness in Northumberland & North Tyneside. 2. Identify **gaps and opportunities in** providing support for people experiencing or at risk of homelessness in Northumberland & North Tyneside. 3. Understand how well support is **currently interlinked/ integrated** within Northumberland and North Tyneside. 4. Understand **extent of trauma-informed approaches in** support for people experiencing or at risk of homelessness in Northumberland & North Tyneside. |
| --- |

**Interview Script**

**Details & Characteristics**

*I’d like to start by asking you a few questions about yourself and the service you are representing.*

1. What is your name?
2. What is the name of your organisation or group?
3. Which of the following best represents the type of organisation or group you work for?
   1. Education
   2. Local government
   3. Health
   4. Charity
   5. Other – please specify:
4. Which geographical areas does your service/support cover:

**Services Supporting People Experiencing Homelessness**

*Thank you. Now I’m going to ask you a few questions on the type of support or service your organisation (or those you commission) provides for people experiencing homelessness.*

1. Roughly, what percentage of your clients’ experience homelessness (including insecure housing, sleeping on a friend’s sofa, rough sleeping, staying in supported housing)?
2. Thinking about the broadest definition of homelessness. What type of homelessness do your clients experience? (e.g., insecure housing, sleeping on a friend’s sofa, rough sleeping, staying in supported housing):
   1. Other – please specify:
3. What type of support is offered by your organisation or group to these clients?
4. Is your service based around planned or unplanned support? If planned, where do you refer for unplanned support?
5. If you could let us know in as much detail as possible, who is involved in providing this support to clients? (for example, staff roles, volunteers, qualifications, training, etc?)
6. What are the operational hours for the service/support provided by your organisation or group?
7. How do these clients access this support? (E.g. referral from GP, walk-in, etc.)
8. Does your service have any criteria or threshold for accessing the service? For example, this could be that your service is only available to females or doesn’t see people that currently consume alcohol.
9. Is the funding for your service short-term or long-term?
10. Who commissions the service? Is it a local, regional, or national commissioning agency?

**Gaps in Service Provision**

*Thank you for telling me more about the support your organisation provides. I’m going to ask some questions to help understand some of the gaps in services for homeless people, and what you think might help to building better networks across services for that wrap-around care for homeless people.*

1. Do you think there are any gaps across services or support for people experiencing homelessness?
2. What might help overcome these gaps?
3. Are there any opportunities across services to provide better wraparound care for those who are homeless?
4. If different services aren’t connecting, why do you think that is?

**Trauma-Informed Care**

Very often people who are homeless will have experienced trauma of some kind, physical or psychological, and there is a recognition now for services to be more trauma informed. So, I’m going to ask you some questions now about exploring opportunities for services to be more trauma-informed.

1. Would you say your service is trauma-informed and why?
2. Does your service provide trauma-informed training to staff, and can you describe a little about what it looks like?
3. Is your workforce offered other training such as psychologically informed environments?
4. Do you think there is a need for more trauma-informed approaches in your service and can you explain why or why not?
5. What could help bring about this trauma-informed approach into your service?

**Relational Questions (Social Network Analysis)**

Thank you for your input so far. I’m now going to ask some questions specifically on the relationships between your service and other services. These questions are structured and may take us a few minutes to get through them.

1. Could you list up to 5 organisations that your service works most closely with – you may have more than 5 but what are the top 5 you work most closely with (thinking about your clients experiencing homelessness)
   1. Organisation 1:
      1. Contact details:
   2. Organisation 2:
      1. Contact details:
   3. Organisation 3:
      1. Contact details:
   4. Organisation 4:
      1. Contact details:
   5. Organisation 5:
      1. Contact details:
2. Does your service make referrals to this organisation? (yes/no)
   1. Organisation 1:
   2. Organisation 2:
   3. Organisation 3:
   4. Organisation 4:
   5. Organisation 5:
3. Does your service receive referrals from this organisation? (yes/no)
   1. Organisation 1:
   2. Organisation 2:
   3. Organisation 3:
   4. Organisation 4:
   5. Organisation 5:
4. Does your service also provide services to clients of this service? (yes/no)
   1. Organisation 1:
   2. Organisation 2:
   3. Organisation 3:
   4. Organisation 4:
   5. Organisation 5:
5. Do your clients receive services from this service? (yes/no)
   1. Organisation 1:
   2. Organisation 2:
   3. Organisation 3:
   4. Organisation 4:
   5. Organisation 5:
