# Supplementary figures and images for: Mapping the organisational network of support for people experiencing homelessness in rural coastal areas of North East England: Results from a mixed-methods multi-sector social network analysis
Source: PLOS Ment Health. 2024 Dec 20;1(7):e0000207. doi: 10.1371/journal.pmen.0000207 (PMC12798162; doi:10.1371/journal.pmen.0000207)

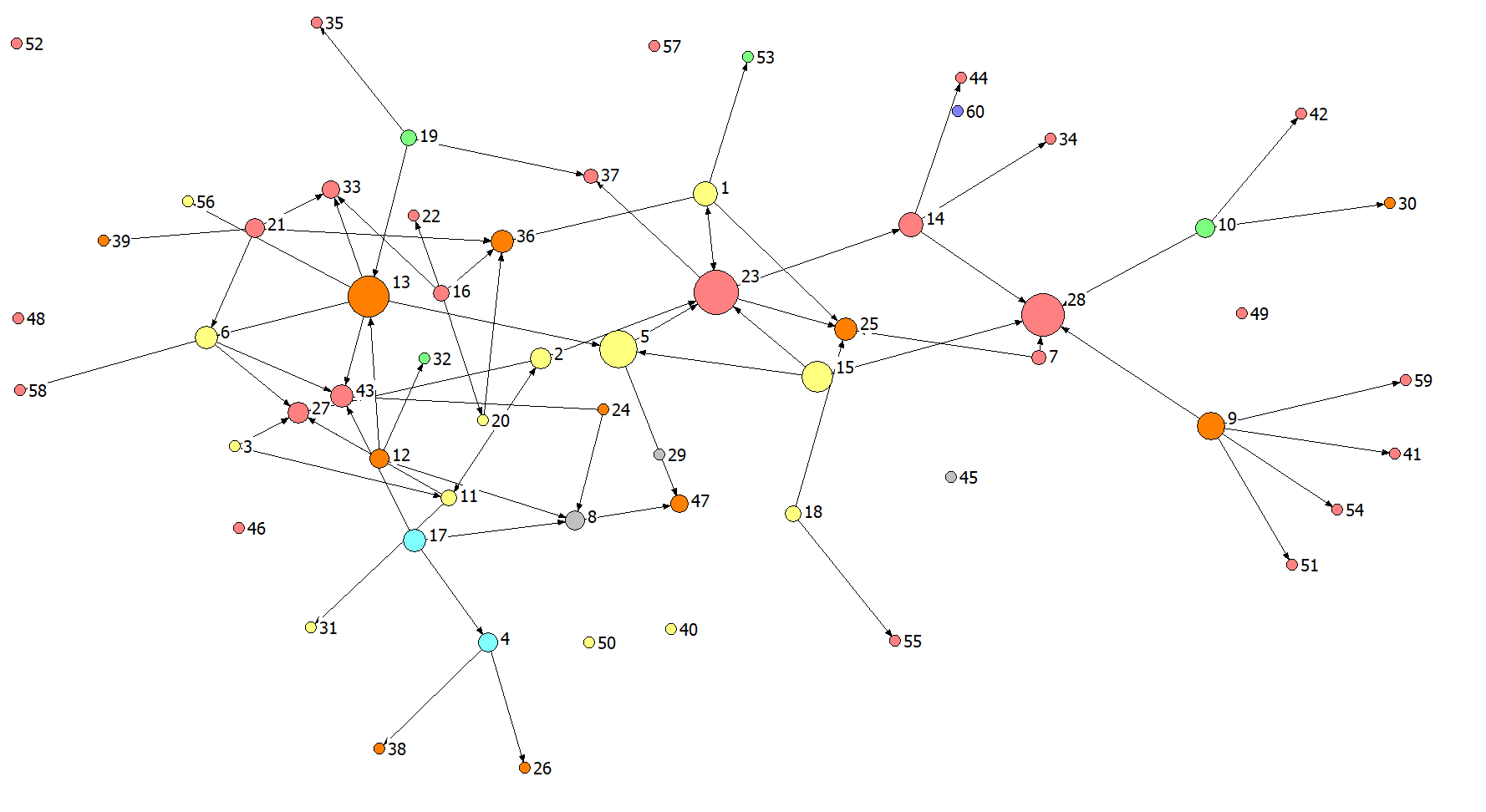

Supplement: S1 Fig — (TIF) [file pmen.0000207.s002.tif]

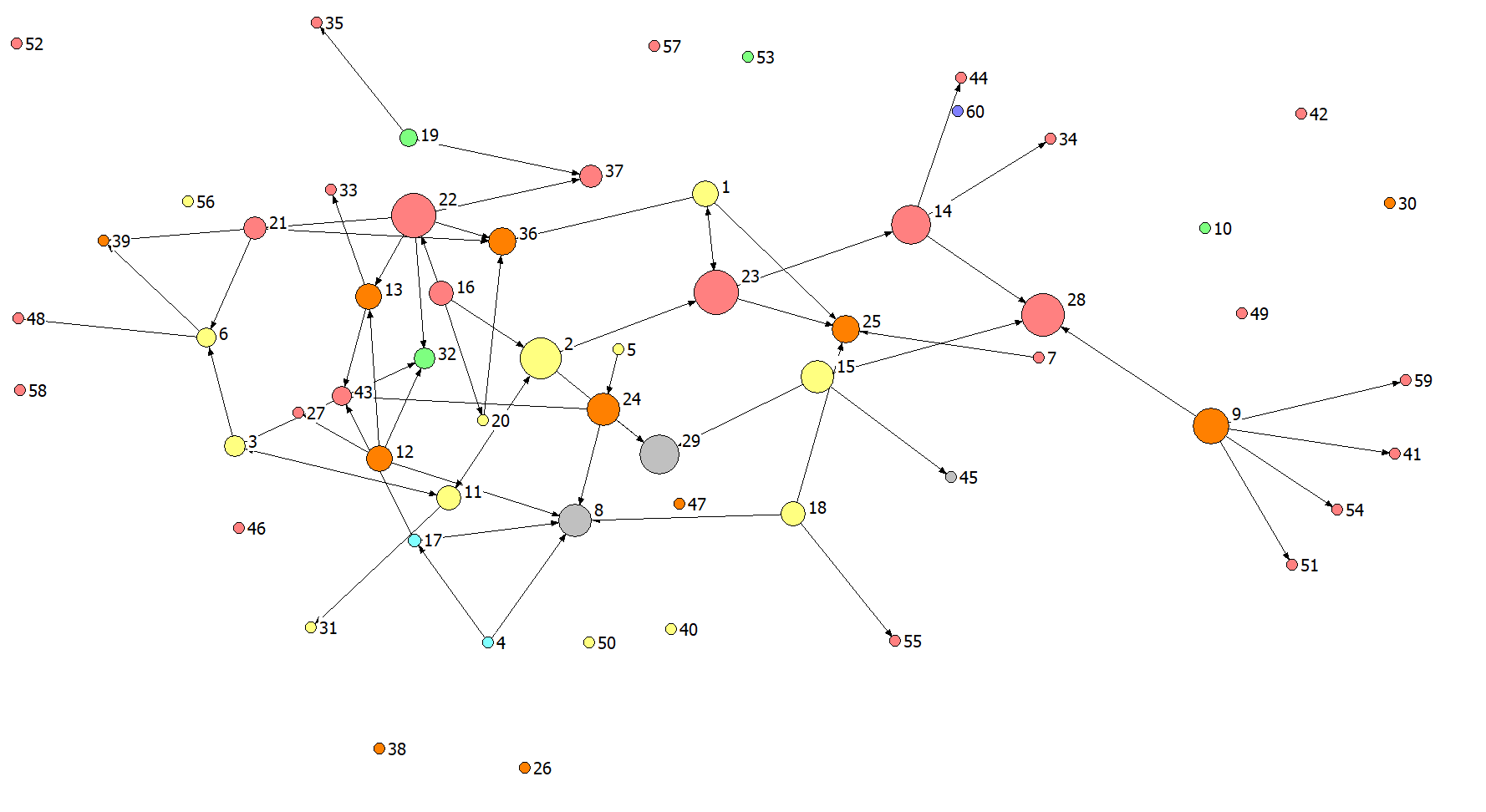

Supplement: S2 Fig — (TIF) [file pmen.0000207.s003.tif]

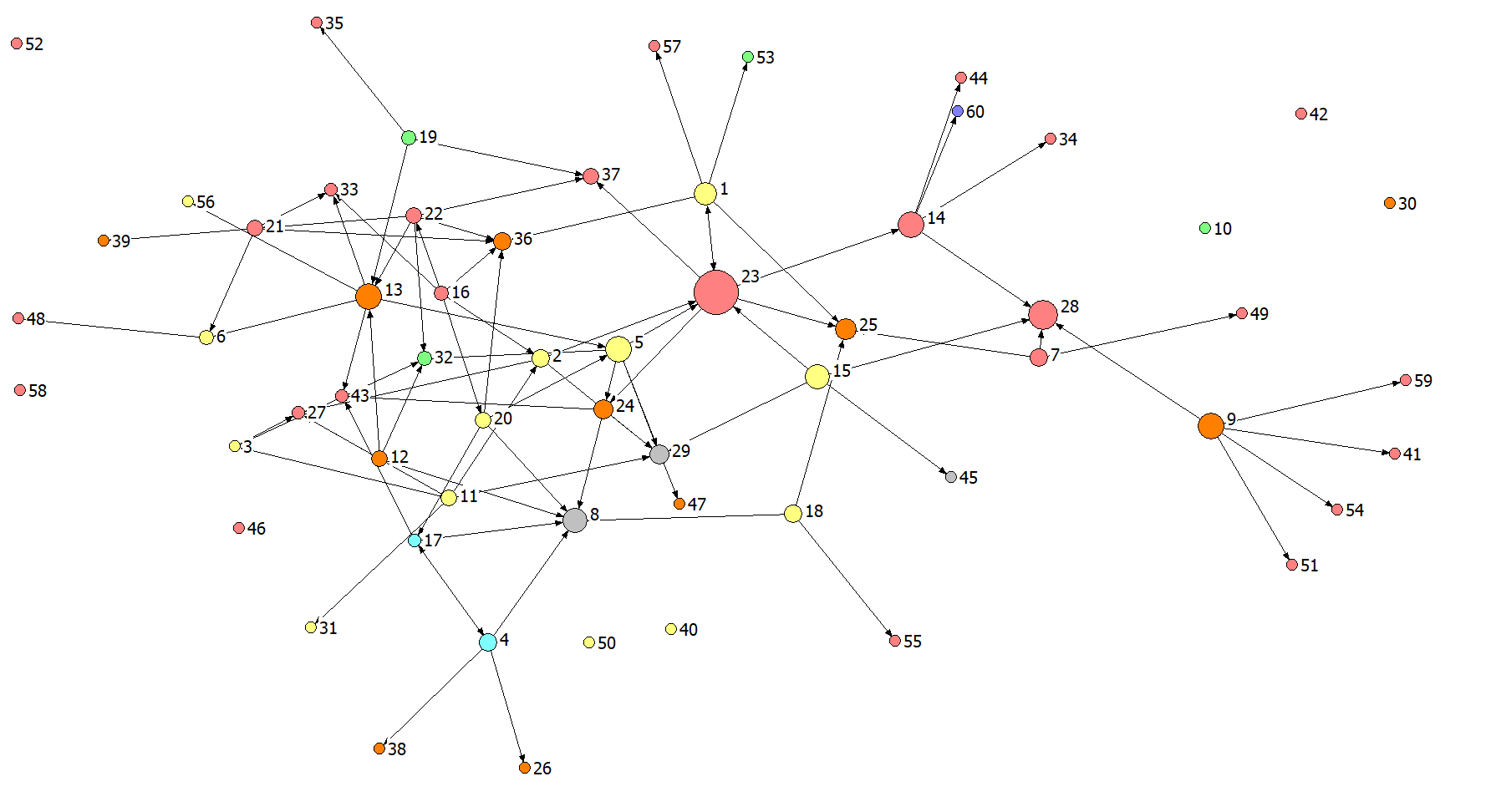

Supplement: S3 Fig — (TIF) [file pmen.0000207.s004.tif]

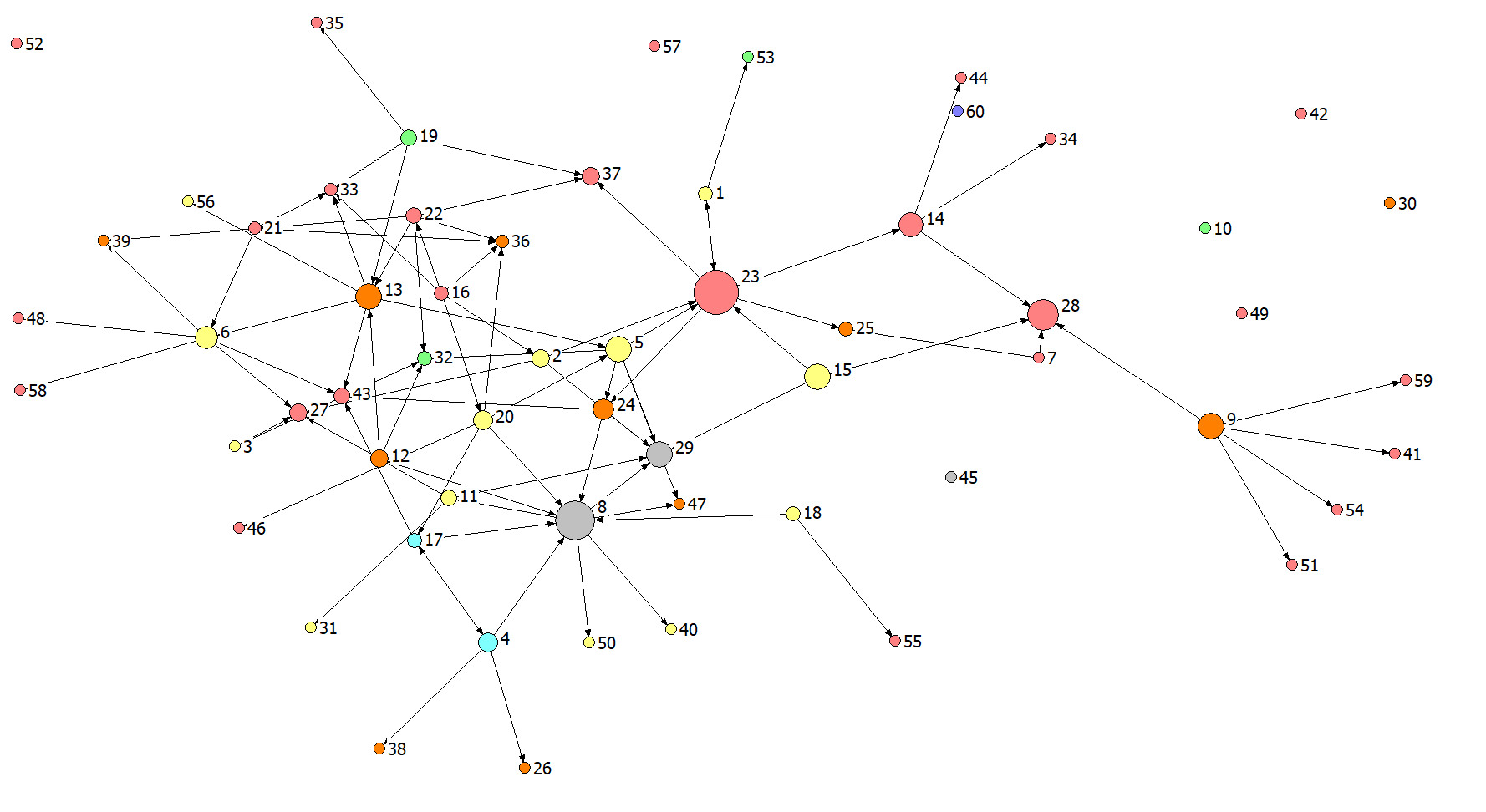

Supplement: S4 Fig — (TIF) [file pmen.0000207.s005.tif]
