# Supplementary material for: Mapping the organisational network of support for people experiencing homelessness in rural coastal areas of North East England: Results from a mixed-methods multi-sector social network analysis
Source: PLOS Ment Health. 2024 Dec 20;1(7):e0000207. doi: 10.1371/journal.pmen.0000207 (PMC12798162; doi:10.1371/journal.pmen.0000207)
Supplement: Attachment — Submitted filename: Response to Reviewers 04 10 2024.docx [file pmen.0000207.s006.docx]

**Response to reviewer comments**

Manuscript ID PMEN-D-24-00149 entitled “Mapping the network of support for people experiencing homelessness in rural and coastal areas of north-east England: Results from a mixed-methods multi-sector social network analysis”

**Authors’ response:** We thank the Reviewers for taking the time to read the manuscript and providing their useful feedback. We have provided a point-by-point response to the comments below.

**Reviewer: 1**
Many thanks for this interesting article. It is on the overlap of 2 important topics which are still each relatively neglected, namely homelessness and rural/coastal care systems. It covers the topic really well, in terms of style of writing and connections to existing literature. Many of the findings resonate with those from the ongoing programme of work led by Michelle Cornes (<https://www.kcl.ac.uk/research/homelessness-research-programme>), which has most recently been focused on discharge from hospital for people experiencing homelessness, including work to understand integrating care in that process (<https://www.emerald.com/insight/content/doi/10.1108/JICA-03-2021-0012/full/html>). A few points for the authors to consider:

**Comment 1:** Ln 65 – “In rural areas, homelessness is often “hidden” in the forms of sofa surfing, temporary accommodation, etc. which is much less visible than homelessness in urban areas …” – these forms of homelessness occur in urban areas as well; might be better to specify “much less visible than street homelessness in urban areas”

**Authors’ response:** We thank the Reviewer for this useful comment. To provide clarity, we have revised the sentence to specifically mention that homelessness in rural areas is much less visible than in urban areas.

**Changes made in the manuscript: Introduction, Pg 3.** “In rural areas, homelessness is often much less visible or “hidden” in the forms of sofa surfing, temporary accommodation, etc. than homelessness (such as street homelessness) in urban areas”.

**Comment 2:** Ln 76 “This often includes physical and mental health” – “often includes”?

**Authors’ response:**

We agree and thank the Reviewer for highlighting this error and have amended the sentence.

**Changes made in the manuscript: Introduction, Pg 4.** “This often includes physical and mental health problems”.

**Comment 3:** More discussion of Northumberland and North Tyneside would be helpful to set the context, particularly for international readers.

**Authors’ response:**

We appreciate the Reviewer's suggestion to provide more context about Northumberland and North Tyneside for international readers. We have added more detail in the Introduction, Methods and Discussion.

**Changes made in the manuscript:**

As suggested by the Reviewer, we have added more context on the geographical areas in the Introduction, Methods and Discussion sections.

**Methods, Pg 6.** “This study employed a mixed methods design to understand the relationships between services for people experiencing homelessness in Northumberland and North Tyneside, rural and coastal areas in North East England. These geographically diverse areas, distributed across almost 2000 square miles, have distinct demographic characteristics. Both have a predominantly white British population (over 95% of residents). Northumberland, primarily a rural county with approximately 325,000 residents, has a higher proportion of older individuals. North Tyneside, comprising rural, coastal, and suburban areas, has a smaller population of approximately 210,000 with a younger median age (36). Data were collected from December 2022 to April 2023 through surveys and semi-structured interviews with staff from selected services that often support people experiencing homelessness (37).”

**Discussion, Pg 24.** "The findings of this study are particularly relevant given the recent increases in homelessness in Northumberland and North Tyneside (45). The sparse network structure and limited interconnectedness among services identified in this study may be exacerbated by the geographical diversity of these areas, which span almost 2000 square miles and include rural, coastal, and suburban settings. The demographic differences between Northumberland (larger, more rural, with an older population) and North Tyneside (smaller, more diverse in landscape, with a younger population) likely contribute to the varied challenges in service provision and coordination across the region.

These contextual factors highlight the importance of developing tailored approaches to address homelessness in rural and coastal areas. The study's findings on the need for trauma-informed care and multiagency collaboration become even more critical when considering these demographic and geographic characteristics, as they highlight the necessity for flexible, context-specific strategies in addressing homelessness in diverse rural and coastal settings.”

**Comment 4:** Recruitment – how did you decide to stop? was a point of ‘saturation’ reached or was the end reached because of project time limits?

If possible also say how many organisations in the final sample were in each of the categories of services you list.

**Authors’ response:** We thank the Reviewer for this question about our recruitment process and sample composition. As the Reviewer suggested, we continued recruitment until a point of saturation was reached in terms of types of services and organisations. We have added information to clarify our recruitment end point and have also now provided a breakdown of our final sample composition.

**Changes made in the manuscript:**

**Methods, Pg 7.** “Recruitment continued until a point of saturation was reached in terms of services or organisations were identified .”

**Results, Pg 11.** “The final sample consisted of: Housing (7), Healthcare (2), Emergency care services (2), Local authority (4 services across two Local Authorities), Criminal justice (1), and Advice and support services (8).”

**Comment 5:** “Local Authority” encompasses a large range of services (e.g. social care, public health, housing, education) – can you say more about which of these were in the network?

**Authors’ response:** We appreciate the Reviewer's suggestion for more specific information about the Local Authority services included in our network. In our revised manuscript, we have provide a more detailed breakdown of the Local Authority services represented in our study.

**Changes made in the manuscript: Results, Pg 11.** “Local Authority services in the network covered a broad range of support areas, including housing services (such as homelessness options teams and emergency housing), social care services, mental health services, poverty alleviation services, and youth support services.”

**Comment 6:** “participants were mostly senior members of staff” does this mean senior frontline staff (consultants, principal social workers etc.) or senior managers not working on the frontline, or both?

**Authors’ response:** We thank the Reviewer for suggestion clarification on the roles of our participants. We have revised the sentence to provide more specific information about the participants' positions.

**Changes made in the manuscript: Results, Pg 11. “**The participants were primarily senior members of staff, including both senior frontline professionals (such as consultants and principal social workers) and senior managers, all of whom were involved in or had knowledge of collaborations with other services.”

**Comment 7:** Strengths and weaknesses – discussion of S&Ws of case study approach ie in depth but might not apply to other localities.

**Authors’ response:** We thank the Reviewer for their suggestion to discuss the strengths and weaknesses of our case study approach. We have added to our "strengths and limitations" section to address this point.

**Changes made in the manuscript: Discussion, Pg 28.** “Our study's focus on Northumberland and North Tyneside represents both a strength and a limitation. As a case study, it allowed for an in-depth examination of support networks for homelessness in these specific rural and coastal areas, providing rich, contextual data. This approach enabled us to uncover nuanced insights about service provision and challenges unique to these localities. However, we acknowledge that the findings may not be directly generalisable to other areas, particularly those with different geographical, demographic, or service landscapes. While the insights gained can inform understanding of rural and coastal homelessness support more broadly, caution should be exercised in applying these findings to significantly different contexts.”

**Comment 8:** Ln 75-6 – it isn’t just the rage of support that people need which is at issue, it is how to organise these to simultaneously address complex ad interacting issues (e.g. homelessness, physical and mental health issues, and substance use) rather than in a sequential way as services often seem to want to do

**Authors’ response:** We thank the Reviewer for this valuable comment regarding the organisation of support services. We have revised our text to incorporate this important point.

**Changes made in the manuscript: Introduction, Pg 4.** “This difficulty is compounded when services are not integrated, requiring individuals to navigate multiple systems independently.”

**Comment 8:** Lns 86-8 unpack a little more about the need for a unified approach to trauma support – why it is important and how being in dispersed services makes it harder.

**Authors’ response:** We thank the Reviewer for their suggestion to elaborate on the importance of a unified approach to trauma support and the challenges posed by dispersed services. We have expanded on this in our introduction to address these points more comprehensively.

**Changes made in the manuscript:**

**Introduction, Pg 4.** “A unified approach to supporting people with experiences of trauma is crucial because it ensures consistency in care, prevents re-traumatisation across different service interactions, and promotes a holistic understanding of an individual's needs. This is particularly important for people experiencing homelessness, who often interact with multiple services and may have complex trauma histories.”

**Introduction, Pgs 4 & 5.** “The geographical spread of services makes coordination more challenging, potentially leading to inconsistent approaches to trauma support. Additionally, the isolation of services in rural and coastal areas may limit opportunities for shared training and knowledge exchange on trauma-informed practices, further complicating the implementation of a cohesive approach."

**Reviewer: 2**
This is an interesting study and a potentially useful application of network analysis methods to an important topic. I think some more work is required to make the analysis more robust, improve the presentation of the methods and findings, and to better understand the implications of the findings and strengthen the discussion.

**Comment 1:** Title – It would be useful to clarify that the study looks at organisational networks. At first read of the current title, it could be assumed that the article focuses on social support networks for homeless individuals.

**Authors’ response:** We appreciate the suggestion and revised the title to explicitly mention that we are examining the organisational network of services.

**Changes made in the manuscript: Title change:** Mapping the Organisational Network of Support for People Experiencing Homelessness in Rural Coastal Areas of North East England: Results from a Mixed-Methods Multi-Sector Social Network Analysis

**Short title change:** Organisational Support for Homelessness in North East England

**Comment 2:** Abstract: Methods:
Brief information on the topics/areas of the surveys and interviews would be helpful. What do the ties in the network represent? What were the topics of interview discussion?
I’m not sure what linkage density is and how it differs from density. Density is a network level metric (one value per network) while degree and betweenness centrality are node level metrics (one value per node), it would be clearer to refer to degree distribution, suggest changing to “distribution of degree and betweenness centrality).
“Thematic analysis of interviews identified support gaps and opportunities for trauma-informed care.” – this reads more like results than methods.

**Authors’ response:** We thank the Reviewer for their feedback on the Methods section of our abstract. We have revised this section to provide brief information on survey and interview topics, clarify what network ties represent, and refine our description of network metrics. We've also removed the mention of specific thematic analysis results from the Methods section.

**Changes made in the manuscript:**

**Abstract, Methods, Pg 2**. “A mixed-methods study was conducted in rural and coastal areas of North East England, using purposive and snowball sampling for recruitment. Surveys collected data on inter-organisational collaborations, referrals, and service provision, identifying up to five key partner organisations for each service. Semi-structured interviews explored service provision for people experiencing homelessness, gaps in service provision, and trauma-informed care practices. Social network analysis was used to map and characterise organisational networks based on survey results, measuring network density, distribution of degree and betweenness centrality, which were used to assess the integration and coordination of services. Thematic analysis was applied to interview data.”

**Comment 3:** The average ties per service isn’t the best measure of decentralisation, you could report degree centralisation or betweenness centralisation, this is the variation in degree between all nodes in the network. If one node has a really high degree, but others low, this is a centralised system.

**Authors’ response:** We appreciate the Reviewer's suggestion regarding measures of decentralisation. We have included both degree centralisation (and now for each service type) and betweenness centralisation measures in our analysis. These measures are reported later in the manuscript, offering insights into the variation of connections across nodes and identifying potential key players in the network.

**Changes made in the manuscript:** As suggested by the Reviewer, we have now reported degree centralisation in the results presented in Table 3.

**Comment 4:** Conclusions mention key players being advice and support services, did the advice and support services have a higher degree or more central position in the network? This is how “key player” is normally measured in network analysis. If “key player” theory is an important conclusion, the results should reflect the key player analysis.

**Authors’ response:** We appreciate the Reviewer's comment about key players in our network. We have included the mean (with minimum and maximum) of degree centrality by service type. We have incorporated these findings into our Results section and revised our discussion of key players to reflect this nuanced understanding. While some advice and support services are indeed the most connected, we now also highlight the important connecting roles of emergency care and criminal justice services based on their high mean degree centrality.

**Changes made in the manuscript: Results, Pg 16.** “Analysis of degree centrality by service type reveals varying levels of connectivity across the network. Advice and support services demonstrate the highest maximum degree centrality (0.131), indicating the presence of some highly connected services within this category. However, emergency care services exhibit the highest mean degree centrality (0.065), closely followed by criminal justice services (0.059). Local authority services show a wide range of connectivity, suggesting diverse roles within the network.”

**Abstract, Pg 3.** Key players emerged across service types, with advice and support, emergency care, and criminal justice services as important intermediaries.

**Discussion, Pg 26.** The social network analysis highlighted the complex roles of key players in the network. While some advice and support services emerged as highly connected actors, the analysis revealed that emergency care and criminal justice services also play crucial connecting roles. These services act as central brokers, connecting different actors and facilitating information and resource exchange, functions crucial for overcoming the unique challenges of service provision in rural and coastal areas.

**Discussion, Pg 26.** The presence of key players across different service types provides diverse opportunities to strengthen the network and improve service provision. By leveraging the influence and connectivity of these key players, there is potential to enhance support in these geographically dispersed communities, recognising the distinct contributions of different service categories.

**Comment 5:** Methods: Recruitment:
Snowball sampling was mentioned. Was this snowballing from the organisations mentioned in the network survey? Were all 60 services in the network invited to take part?
Were the focus groups people from multiple organisations, or several people from the same organisation only?

**Authors’ response:** We thank the Reviewer's questions about our recruitment process. We have revised our Methods section to clarify the snowball sampling process, the invitation of services, and the composition of focus groups. Snowball sampling was based on recommendations from initially recruited organisations. A total of 60 services were identified in the network analysis. Representatives from 24 services participated in interviews. The remaining 36 either did not respond or were unable to participate despite our attempts to include them. The focus groups consisted of participants from the same organisation. We have integrated this information into our Methods section to provide a more comprehensive picture of our recruitment process and its challenges.

**Changes made in the manuscript:**

**Methods, Pg 7.** “Additionally, snowball sampling was employed to identify other services, based on recommendations from initially recruited organisations. Of the 60 services identified in the network, 24 actively participated in the study by attending an interview. Attempts were made to invite all nominated services, but 36 either did not respond or were unwilling to participate due to various reasons such as time constraints or lack of interest in the study.”

**Methods, Pg 7.** “…with two participants each from the same organisation”

**Comment 6:** Survey:
Please specify the verbatim name generator text used to elicit the names of the five services. Also add the verbatim text of the additional questions. Network analysis studies rely on tailored questions rather than standardised items, so providing the verbatim text promotes replicability and transparency. It’s also fine to report whether the questions were similar or exact replications of the name generators used in 33,35,36.

**Authors’ response:** We thank the Reviewer for their suggestion to include the verbatim text of our survey questions. We have revised our Methods section to include the exact wording of the name generator question and the four additional questions used to explore the nature of inter-organisational relationships. These questions were adapted from previous studies (34, 37, 38) but tailored to our specific research focus.

**Changes made in the manuscript:**

**Methods, Pgs 7 & 8.** The verbatim text of the name generator question was: “Could you list up to 5 organisations that your service works most closely with?” Following this, four additional questions were asked about each named organisation:

1.“Does your service make referrals to this organisation?” (yes/no)

2.“Does your service receive referrals from this organisation?” (yes/no)

3.“Does your service also provide services to clients of this service?” (yes/no)

4.“Do your clients receive services from this service?” (yes/no)

These questions were designed to capture the nature and direction of relationships between organisations”

**Comment 6:** Semi-structured interviews/focus groups:
Again, report detail of the semi structured prompts. It would be good practice to add an appendix with the full interview guide.

**Authors’ response:** We have revised our Methods section to include a summary of the main interview topics. We have included the full interview guide in the Appendices.

**Changes made in the manuscript: Methods, Pg 8.** “Semi-structured interviews and focus groups were conducted to gain qualitative insights into service relationships and effective service provision. The interview guide covered three main topics:

1. Services Supporting People Experiencing Homelessness: This included questions about client demographics, types of support offered, service accessibility, and operational details.
2. Gaps in Service Provision: Participants were asked to identify gaps in services, potential solutions, opportunities for improved wraparound care, and barriers to service connection.
3. Trauma-Informed Care: This section explored current trauma-informed practices, staff training, the need for additional trauma-informed approaches, and barriers and facilitators to implementation.”

**Methods, Pg 8.** “The full interview guide can be found in Appendix A”

**Comment 7:** Did the survey and the interview happen at the same time? Did any of the interview relate to discussion of the five services named in the survey explicity?
Was there – or in a revision, could there be - any integration of the SNA and qual data? For example, did the most central services talk about different issues / aspects of collaboration than the more peripheral services?

**Authors’ response:** We thank the Reviewer for these suggestions. The interviews and surveys were conducted during the same meeting, with the interview preceding the researcher-led survey. The interview content did not explicitly relate to the services later named in the survey.

Regarding the integration of SNA and qualitative data, we carefully considered this suggestion. However, due to the design of our study and the nature of the data collected, it was not possible to combine these data sets in a meaningful way that would allow for a comparison of central and peripheral services' perspectives. Our qualitative data was not structured to capture information that could be directly mapped onto network positions.

**Changes made in the manuscript: Methods, Pg 7.** The interviews and surveys were conducted during the same meeting with each participant. The interview was conducted first, followed by a researcher-led completion of the survey

**Comment 8:** “formal consent” – I think this should be informed consent.

**Authors’ response:** Thank you for bringing this typographical error to our attention. We have made this change.

**Changes made in the manuscript: Methods, Pg 6.** “all participants in this study provided informed consent”

**Comment 9:** Data analysis:
It would be useful to give information on the total number of survey participants there were, and how many unique new services they mentioned.

**Authors’ response:** In responding to Comment 5 above, we have added this information into the Methods section of the paper.

**Changes made in the manuscript:**

**Methods, Pg 6 & 7.** “Additionally, snowball sampling was employed to identify other services, based on recommendations from initially recruited organisations. Of the 60 services identified in the network, 24 actively participated in the study by attending an interview. Attempts were made to invite all nominated services, but 36 either did not respond or were unwilling to participate due to various reasons such as time constraints or lack of interest in the study.”

**Methods, Pg 7.** “…with two participants each from the same organisation”

**Comment 10:** Density is the terminology used to refer to the network metric that is the total observed ties divided by possible ties. Linkage density was Nicaise’s terminology for referring to density, but these aren’t different measures.
Centralisation and centrality are different measures, one a node level, one a network level. Both are discussed in the results so both should be defined here.

**Authors’ response:** We thank the Reviewer's for their comments on network terminology. We have replaced "linkage density" with "density" throughout the manuscript, now defining it as the total observed ties divided by possible ties. We've also added definitions for centralisation and centrality to clarify the distinction between these concepts.

**Changes made in the manuscript:** We have replaced "linkage density" with "density" throughout the manuscript, now defining it as the total observed ties divided by possible ties. We've also added definitions for centralisation (a network-level measure of power concentration) and centrality (a node-level measure of a service's importance) to clarify the distinction between these concepts.

**Comment 11:** The bullet point on linkage density is incorrect and should be deleted. There is an upper limit on the total number of possible connections - N (N -1) / 2. It’s not usual to report this, instead report the density which is the observed / the total possible.

**Authors’ response:** We thank the Reviewer for pointing out this error. We have removed the incorrect information about linkage density.

**Changes made in the manuscript:** As per our response above in Comment 10, we now report only the density, which is calculated as the observed connections divided by the total possible connections.

**Comment 12:** I’d suggest looking up some key papers with the definition of betweenness centrality and the other metrics defined in the methods. Betweenness is the total number of shortest paths the node sits on, it can be normalised to be between zero and one. A high betweenness node need not be the ‘sole’ bridge, there may be many other bridges that are more, less, or equally central. Consider adding a citation for information on these metrics, both to verify the calculations and to provide information for the interested reader to look into for themselves.

**Authors’ response:** We have updated our definition of betweenness centrality to more accurately reflect its meaning and interpretation. We've also added relevant citations to all our network metric definitions.

**Changes made in the manuscript: Methods, Pg 10.** “Betweenness centrality: A measure of how often a node appears on shortest paths between other nodes in the network. It ranges from 0 to 1 when normalised. A score of 0 means the service never acts as a bridge in the shortest path between other services, while higher scores indicate the service more frequently acts as a bridge. High betweenness centrality doesn't necessarily mean a service is the sole bridge, as there may be multiple important connectors in the network. (41, 43)”

**Comment 14:** The authors have stated that data won’t be shared because it contains potentially identifiable information. I think that the network data relating to Figure 1 i.e. the adjacency matrix and service type, with numbers instead of actual service names could be made available without risking any disclosure of personal information. This could be made available without the associated qualitative data.

**Authors’ response:** We thank the Reviewer for their suggestion regarding data sharing. To balance transparency with participant confidentiality, we have revised our data availability statement. The adjacency matrix and service types for Figure 1, using numerical identifiers, will be available upon reasonable request to the corresponding author. Other data remain confidential due to their sensitive nature.

**Changes made in the manuscript: Updated data availability statement:** Due to the sensitive nature of the data and to protect participant confidentiality, most of the data generated and/or analysed during the current study are not publicly available. However, the adjacency matrix and service types, using numerical identifiers instead of service names, are available from the corresponding author upon reasonable request. All other data, including qualitative information, remain confidential as they contain potentially identifiable information. Summaries of the data are available from the corresponding author upon reasonable request.

**Comment 15:** Whole network characteristics:
If 24 services were asked to name up to five services each, that implies there would be 120 possible ties. In this section it appear there are 174 receive service ties. I’m presuming this is because there were more than one name generator with the option of five responses, or there were more than five points on the name generator but this needs to be clarified in the methods.

**Authors’ response:** We thank the Reviewer for this insightful observation. The higher number of ties reflects our method of counting both incoming and outgoing relationships for each service pair across four relationship types. We have clarified this approach in the Methods and Results sections to ensure clarity in our network analysis reporting.

**Changes made in the manuscript: Results, Pg 12.** “There were 198 ties out of a possible 240 when examined as a unique connection”.

**Comment 16:** It would be useful in table 1 to report the “flattened” network. There are 602 ties in total, but that includes double counting of ties between the same pair of services. How many ties are there if you count any type of tie between two services as the presence of a tie, and otherwise no tie?

**Authors’ response:** We thank the Reviewer for this suggestion. Table 1 has been revised to include both the total number of directed ties (602) and the number of unique connections (198). There were 42 instances of there being no ties across the four relations.

**Changes made in the manuscript:** Updated Table 1 to include both the total number of directed ties and the number of unique service connections in the flattened network.

**Results, Pg 12.** “There were 198 ties out of a possible 240 when examined as a unique connection. “

**Comment 17:**There is no mention of the directionality of the network. Service A may nominate Service B as providing services, but Service B may also nominate A as providing services. What are the implications of ignoring this directional information and considering any report as presence of a tie (the weak symmetrisation rule), what would happen if you only considered a tie as present if both A and B report that tie as present (the strong symmetrisation rule). I would tend to opt for the weak rule, but some consideration of this in the data would be informative. Perhaps reporting the dyad census or triad census would give some descriptive information on this.

**Authors’ response:** We thank the Reviewer for their comments on network directionality. We've clarified our use of the weak symmetrisation rule in the Methods section and conducted a triad census as suggested. The triad census methodology, results, and implications have been added to the Methods, Results, and Discussion sections respectively. We've also considered and added to the implication section how the weak symmetrisation rule affects our interpretation of the network structure.

**Changes made in the manuscript:**

**Methods Pg 9.** “This analysis employed the weak symmetrisation rule, which considers ties present if reported by either service. This approach was chosen to capture all reported relationships.”

**Methods, Pg 10**. “A measure to analyse the network's micro-structure. This method counts and categorises all three-node subgraphs (triads), revealing patterns of connectivity and reciprocity. It identifies common relationship structures and calculates the network's transitivity, measuring the tendency for nodes to form closed triangular connections.

**Results, Pg 14.** “A triad census was conducted to examine the network's structure. The majority of triads were empty subgraphs (28,864), followed by single directed edge triads (4,786). Mutual connections between two vertices were observed in 203 triads. The network's transitivity was 0.129, indicating a low level of triadic closure.”

**Discussion, Pg 25.** “The triad census results and low transitivity (0.129) reveal a network structure with limited interconnectedness, highlighting significant opportunities for enhancing service integration. This sparse connectivity suggests that many potential relationships between services remain unexplored or undeveloped.

**Discussion, Pgs 27 & 28.** “The use of the weak symmetrisation rule in our analysis allowed us to capture a broader range of reported relationships. This approach may have implications for how we interpret the density and structure of the network, potentially showing more connections than a strong symmetrisation rule would have revealed.”

**Comment 18:** Lines 233 – 237 – as mentioned above, the total number of ties is relative to the number of nodes in question, so it’s more useful to consider the density.

**Authors’ response:** We thank the reviewer for emphasising the importance of network density. Our analysis includes both the total number of ties and network density.

**Changes made in the manuscript:** We have retained the total number of ties but have emphasised the discussion on density in the discussion section.

**Comment 19:** Table 2 mentions all-degree but this wasn’t defined in the methods section. It’s more common terminology to refer to total degree rather than all-degree. Instead of reporting the highest number of ties and which service type it was for, it would be more useful to report the minimum, mean and maximum values for each type of service as additional rows in the table.

**Authors’ response:** We thank the Reviewer for this comment. We have replaced "all-degree" with "average ties per service" for clarity and accuracy.

**Changes made in the manuscript:** We've updated Table 2 to include minimum, mean, and maximum number of ties for each service type, providing a more comprehensive view of connectivity across service types.

**Comment 20:** “Intra-and inter-cluster linkage density” reports the number of intra ties, but not the number of inter-group ties. It may be more informative to report a mixing matrix, which shows the number of ties from one group to each other group. This could be normalised by reporting percentages as well as the number of ties. I’m not sure if UCInet does this automatically, but it can be done in R, see page 11 for an example.
<https://cran.r-project.org/web/packages/ergm/vignettes/ergm.pdf>

I know R isn’t the easiest software to work with, but if you can import your 60*60 matrix into R and assign the service type attribute to the nodes, then the tutorial above could produce this for you.

**Authors’ response:** We thank the Reviewer for their suggestion to include a mixing matrix for a more comprehensive view of inter-group ties. We recognise the potential for more detailed inter-group analysis in future research. We have noted this in our discussion section as an important direction for future studies, which could employ advanced network analysis techniques to further explore the nuances of inter-service relationships in homelessness support networks.

**Changes made in the manuscript:**

**Discussion, Pg 29.** While our study provides insights into the overall network structure, we recognise that a more detailed analysis of inter-group ties, such as through mixing matrices, could offer additional nuanced understanding of the relationships between different service types. Future research could benefit from employing advanced network analysis techniques, such as mixing matrices, to provide a more comprehensive view of inter-service relationships. This could reveal patterns of interaction between different service types and potentially inform strategies for enhancing cross-sector collaboration in homelessness support networks.”

**Comment 21:** I think it would be worth reporting the min, mean, maximum betweenness centrality by each service type in the tables, as their interpretation becomes important in the level of coordination section.

**Authors’ response:** We thank the Reviewer for this comment. We have reported min, mean and max for degree centrality for each service type.

**Changes made in the manuscript:** Added the degree centrality for each service type in Table 3.

**Comment 22:** “Advice and Support Services primarily acting as liaisons” – definition of a liaison hasn’t been given before now, nor what the different types of brokerage roles are. Same comment applies to table 3. I imagine this table would be a useful place for the min, mean, max betweenness scores.

**Authors’ response:** We thank the Reviewer for this suggestion. We have addressed this by adding a description of brokerage roles in the methods section. This includes definitions for coordinator, gatekeeper, representative, consultant, and liaison roles.

**Changes made in the manuscript: Methods, Pgs 10 & 11**

“Brokerage roles: A classification of how a node connects otherwise disconnected groups within a network. Brokerage roles help identify services that play crucial intermediary functions in the network, facilitating information flow and resource exchange between different sectors or service types. Five main brokerage roles are typically identified:

Coordinator: Connects members of its own group

Gatekeeper: Controls information flow from outside to inside its group

Representative: Controls information flow from inside to outside its group

Consultant: Connects two members of the same group (different from its own)

Liaison: Connects members of two different groups, neither being its own”

**Comment 23:** I would tone down the discussion of density as a measure of sparseness. A density of 2% is very common in social networks, because there simply aren’t enough hours in the day for everyone connect with everyone else. Also, the maximum density is limited if each organisation nominates a maximum of five other orgs.

**Authors’ response:** We thank the Reviewer for their insight regarding the interpretation of network density. We have revised our discussion to provide more context for the observed density and to avoid overemphasising sparseness.

**Changes made in the manuscript:**

**Discussion, Pg 25. “**The network density of 0.029 indicates that 2.9% of possible ties are realised, which, given the geographical dispersion and diverse nature of services in rural and coastal areas, suggests a level of connectivity that may present both challenges and opportunities for service coordination. “

**Comment 24:** Figure 1:
More elaboration on the Figure in the text would be helpful to help people interpret the structure i.e. talk about there being a large group of red orgs to the left, with the grey and blue services connected; while to the right there a few less well connected red orgs. All but one of the green orgs tend are on the periphery, while the yellows are often more central.
It’s confusing to encode node type with different shapes and colours, the node shapes have different surface areas, this means that the size of the nodes are harder to compare for the same level of connectivity. It would be better to have all nodes the same shape (circle is most common) and just use colour for node type and size for betweenness centrality.
There are many red nodes in the graph, but red is not listed in the figure legend.

**Authors’ response:** We appreciate the Reviewer's suggestion for a more detailed description of Figure 1. We have expanded our explanation of the network structure in the Results section, providing a comprehensive overview of the distribution and positioning of different service types. We've also added an interpretation of these observations in the Discussion section, relating the network structure to potential roles and involvement levels of various services in supporting people experiencing homelessness.

**Changes made in the manuscript:**

**Results, Pg 16.** “Fig 1 shows the graphical representation of the network, known as a sociogram in social network analysis. The sociogram displays all relations (connections) across the four types of ties measured. Nodes represent services, with their colour indicating service type and size proportional to betweenness centrality. All nodes are depicted as circles to ensure comparability. “

**Results, Pg 17.** “The network structure reveals that advice and support services (pink nodes) are the most numerous and distributed throughout the network, suggesting their widespread involvement in supporting people experiencing homelessness. Housing services (yellow nodes) occupy central positions, appearing to play a crucial role in connecting different parts of the network. Local authority services (orange nodes) are scattered throughout, often connecting to multiple service types, indicating their broad involvement across the support network. Criminal justice services (grey nodes), though few, are positioned centrally, suggesting important connecting roles. Healthcare services (green nodes) and emergency care services (light blue nodes) are fewer in number and tend to be on the periphery, potentially indicating more specialised or less frequent involvement. A single 'other' service (purple node) is present, connected to the network but not central”

**Discussion, Pg 25.** “The distribution of services in the network suggests varying levels of involvement in supporting people experiencing homelessness. The central positions of housing and some local authority services, coupled with the widespread distribution of advice and support services, may indicate their key role in coordinating support efforts. The peripheral position of healthcare and emergency services could suggest more specialised or less frequent involvement in the network. “

**Comment 25:** It would be helpful in an online appendix to see the plots for each type of tie. Referral and service provision are different forms of interaction. The former requires very little investment, while the latter requires much stronger collaborative work. In this sort of system, the density and structure of the referral network may be much higher than the collaboration network, and the implications of being a broker in a referral network are very different than being a broker around co-delivery of work. It would be worth unpacking this a little more, and if the structure and findings differ comparing the aggregated and the service-only network, to discuss the implications.

**Authors’ response:** We appreciate the Reviewer's suggestion for a more detailed analysis of different tie types. We have created separate network visualisations for each type of tie and included these in an online appendix as recommended. However, a comprehensive comparison of network measures for each tie type is beyond the scope of our current study. We acknowledge this as a limitation and have added it as a suggestion for future research in the Discussion section.

**Changes made in the manuscript:**

**Results, Pg 16.** Sociograms displaying the four relations separately can be found in appendix B, C, D and E.

**Discussion, Pgs 28 & 29.** “While we analysed the aggregate network of all tie types, future research could benefit from a more detailed comparison of network measures for different types of relationships (e.g., referrals vs. service provision). Such analysis could provide insights into how different forms of interaction shape the overall support network for people experiencing homelessness in rural and coastal areas. Future studies could explore how the structure and implications of referral networks differ from service provision networks in homelessness support systems, potentially revealing varying levels of collaboration and investment across different types of inter-service relationships.”
