# Supplementary material for: Mapping the organisational network of support for people experiencing homelessness in rural coastal areas of North East England: Results from a mixed-methods multi-sector social network analysis
Source: PLOS Ment Health. 2024 Dec 20;1(7):e0000207. doi: 10.1371/journal.pmen.0000207 (PMC12798162; doi:10.1371/journal.pmen.0000207)
Supplement: Attachment — Submitted filename: Response to Reviewers_PLOSmentalhealth_14 11 2024.docx [file pmen.0000207.s007.docx]

**Response to reviewer comments**

Manuscript ID PMEN-D-24-00149 entitled “Mapping the network of support for people experiencing homelessness in rural and coastal areas of north-east England: Results from a mixed-methods multi-sector social network analysis”

**Authors’ response:** We thank the Reviewers for reviewing our revised manuscript and providing these additional helpful comments. We believe these changes have further improved the clarity, technical accuracy, and overall quality of the manuscript. The Reviewers' comments have been invaluable in helping us refine our work.

We have responded to and addressed each comment as detailed below.

**Reviewer: 1**
Thanks for this. I think the authors have done a great job in responding to all comments from the reviewers.

**Authors’ response:** We thank the Reviewer for their positive response.

**Reviewer: 2**
This is a strong revision, just a few minor suggestions / clarifications. I hope the authors continue to use network analysis in future, and consider presenting their work, obtaining further training, and developing new collaborations around networks and health at relevant conferences e.g. https://sunbelt2025.org/

**Comment 1:** The table mentions Intra-and inter-cluster density but only the intra cluster density is included. The authors opted not to perform the mixing matrix so the reference to inter-cluster ties should be dropped and noted that cross-cluster ties is something for future analysis.

**Authors’ response:** We thank the Reviewer for this helpful observation regarding the table's presentation of cluster density. We agree that since we did not perform a mixing matrix analysis, the reference to inter-cluster density should be removed.

**Changes made in the manuscript:** In Table 2 (**Results section page 15), w**e have removed "and inter-" from the table heading "Intra-and inter-cluster density"

**As suggested, in the Discussion section (page 29, paragraph 1**), we have edited the text related to this point in the implications section. Edited text as follows:

"While our study provides insights into the overall network structure, we recognise that a more detailed analysis of inter-group ties and cross-cluster relationships, such as through mixing matrices, could offer an additional nuanced understanding of the relationships between different service types. Future research could benefit from employing advanced network analysis techniques, such as mixing matrices, to provide a more comprehensive view of inter-service relationships. This could reveal patterns of interaction between different service types and potentially inform strategies for enhancing cross-sector collaboration in homelessness support networks.”

**Comment 2:** The next table states Minimum, mean and maximum of degree centrality by type of service

The values in the table are decimal points, degree centrality is normally presented as the whole number of connections, are these values normalised? For degree, it’s better not to normalise as the number of ties has a natural interpretation no matter how large or small the network. It’s better to normalise for betweenness because its value is dependent on the number of nodes in the network.

**Authors’ response:** We thank the Reviewer for this important technical observation about the presentation of degree centrality. We agree that raw counts of ties provide a more natural interpretation for degree centrality, while normalisation is more appropriate for betweenness centrality due to its dependence on network size. We accept this suggestion and have updated the values accordingly in the Results.

**Changes made in the manuscript: In the Methods section, on page 10,** we have updated the description of degree to include raw counts: “Raw counts are used rather than normalised values to provide a natural interpretation of the number of connections each service maintains”

**As suggested, in Table 3 in the Results Section (page 17)**, we have updated the results as follows:

- Updated the degree centralisation from 0.135 to 6 to represent the non-normalised value
- Revised all degree centrality values (minimum, mean, and maximum) to show raw counts rather than normalised proportions as follows:
- Updated the interpretation in the Results section to read: "Table 3 presents the social network measures of linkage. The betweenness centralisation value of 0.058 and degree centralisation value of 6 suggest a decentralised structure with no single service dominating the network.
